# Validation of the multiple sclerosis diagnosis in the Norwegian Patient Registry

**DOI:** 10.1002/brb3.1422

**Published:** 2019-10-04

**Authors:** Espen Benjaminsen, Kjell‐Morten Myhr, Nina Grytten, Karl Bjørnar Alstadhaug

**Affiliations:** ^1^ Department of Neurology Nordland Hospital Trust Bodø Norway; ^2^ Institute of Clinical Medicine University of Tromsø Tromsø Norway; ^3^ Department of Clinical Medicine University of Bergen Bergen Norway; ^4^ Department of Neurology Haukeland University Hospital Bergen Norway; ^5^ Department of Neurology Norwegian Multiple Sclerosis Competence Centre Haukeland University Hospital Bergen Norway

**Keywords:** epidemiology, health registries, multiple sclerosis, validation

## Abstract

**Background:**

Health registries may yield important data for epidemiological studies. However, in order to be a valuable source for information, the registered data have to be correct.

**Objectives:**

The aim of the study was to validate data from the Norwegian Patient Registry (NPR) regarding multiple sclerosis (MS).

**Materials and Methods:**

We obtained data on individuals residing in Nordland County and registered with a MS diagnosis in the NPR or in local hospital records. The NPR data included a unique 11‐digit personal identity number that made it possible to identify the individuals medical records. For each individual registered with MS in the NPR, the hospital record was scrutinized in order to confirm or rule out the diagnosis.

**Results:**

In Nordland County, 657 individuals had MS 1 January 2017. Of these, 637 were recorded with a correct diagnosis of MS in the NPR, while 59 were recorded incorrectly. Incorrect registration was due to a diagnosis that did not fulfill the diagnostic criteria, later investigation had ruled out MS or it was an error in the diagnostic code registration process. Twenty individuals were not registered with MS in the NPR. These were patients who received their diagnosis before data in the NPR were person identifiable (before 2008), and who later had no MS‐registered contact with public specialist healthcare services. The sensitivity is 0.97, and the positive predictive value is 0.92.

**Conclusion:**

Data from the NPR gave a good estimate of the occurrence of MS, but nearly one in 10 registered diagnoses was not correct.

## Graphical abstract

Health registries can be a valuable source of data for epidemiological studies, but the gain depends on the quality and reliability of the data. We validate multiple sclerosis data from the Norwegian Patient Registry (NPR). Data from NPR give a fair estimate of the real prevalence of MS.

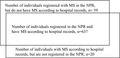

## 1 INTRODUCTION

Knowledge of the epidemiology of diseases can give clues to understanding the etiology and risk factors of diseases. It is also important in healthcare planning.

Health registries can be a valuable source of data for epidemiological studies. There are 18 mandatory national health registries in Norway (Norwegian Institute of Public Health, 2016). These are priceless for health‐related research and innovation, and have provided answers to important medical questions (Håberg et al., 2013). However, the utility of the registries depends on the quality and reliability of the collected data.

One of these registries is the Norwegian Patient Registry (NPR). NPR is a nationwide Norwegian health registry run by The Norwegian Directorate of Health. The registry was established in 1997, and the information is person identifiable from 2008. Whenever a patient is treated at a hospital or a private practice specialists with public reimbursement, the given diagnoses with the corresponding International Statistical Classification of Diseases and Related Health Problems 10th Revision (ICD‐10) codes are mandatorily reported to the NPR. By application, researchers may get access to data from the registry.

Furthermore, all Norwegian citizens have a unique 11‐digit personal identity number, an identity designation retained the whole life. This number is included in every hospital record and is linked to a unique number in the NPR.

With access to detailed hospital records, we aimed to validate data in the NPR regarding MS in Nordland County.

## 2 METHODS

### 2.1 Study population

According to Statistics Norway (Statistics Norway), the population in Nordland County was 242,866 (123,108 men, 119,758 women) January 1, 2017.

Nordland County is in the northern part of Norway and includes the regions of Helgeland, Salten, Ofoten, and the islands of Lofoten and Vesterålen. The public health services for diagnosing and treating MS include the Department of Neurology at the Nordland Hospital in Bodø, and the neurological outpatient services at the hospitals in Mosjøen (Helgeland) and Stokmarknes (Vesterålen). Neurological patients living in the very north of the county (Ofoten) are mostly served by the hospital in Tromsø (Troms County), and patients in the south of the county may be referred to the hospitals in the neighboring county to the south.

### 2.2 Diagnostic criteria

The diagnosis of MS was based on the criteria of Poser (Poser et al, 1983) or McDonald (McDonald et al., 2001; Polman et al., 2011). Individuals were included in the present study if they fulfilled at least one of these criteria.

### 2.3 Case ascertainment

From a previous study, we have detailed knowledge of individuals with MS in Nordland County from 1970 to 2010 (Benjaminsen, Olavsen, Karlberg, & Alstadhaug, 2014). In the present study, we expanded the scope to include data on all patients as of 1 January 2017. In addition to the numbers from the hospital in Bodø, we requested data from the neurological outpatient clinics in Stokmarknes and Mosjøen, Nordland County, and from the hospitals in the neighboring counties, Tromsø to the north, and Namsos and Trondheim to the south.

From the NPR, we received extracted data for all individuals with a diagnosis of G35 (ICD 10) from Nordland County and for all patients with G35 who had had address in Nordland recorded in the period from January 1, 2008, to January 1, 2017.

We validated the MS diagnosis by scrutinizing the hospital records, of which two of the authors (EB, KBA) had full access.

### 2.4 Statistics

The true number of individuals with MS was determined by counting and ascertain all subjects identified in the hospital records who fulfilled the criteria for MS in Nordland County per January 1, 2017. A true positive (TP) was an individual with an MS diagnosis registered in NPR with a validated MS diagnosis based on the hospital records. A false positive (FP) was registered in the NPR, but did not fulfill the criteria for MS. A false negative (FN) was not registered in the NPR, but still fulfilled the criteria for MS. A true negative (TN) was without MS and not registered with MS in the NPR (Table 1). We calculated the sensitivity (TP/(TP + FN)), specificity (TN/(TN + FP)), the positive predictive value (TP/(TP + FP)) and the negative predictive value (TN/(TN + FN)). Cohen's kappa, where the value 0 is agreement equivalent to chance and 1 is a perfect agreement, was calculated to compare the data from the NPR with the true number of individuals with MS. Statistical analyses were performed by the use of Microsoft Excel for Windows 7 and IBM SPSS Statistics version 25.

**Table 1:** Cross‐table indicating the true‐positive, false‐positive, false‐negative and true‐negative values of individuals with or without multiple sclerosis (MS) registered or not registered in the Norwegian Patient Registry (NPR)

	Confirmed MS according to hospital records		SUM
	Yes	No	
Registered MS in the NPR			
Yes	True positive	False positive	Total registered in NPR
No	False negative	True negative	Total not registered in NPR
SUM	Total with MS	Total without MS	Total population

### 2.5 Ethical approval

The study was approved by the Regional Committee for Medical and Health Research Ethics (Rek Nord 2016/1531).

## 3 RESULTS

From the NPR we received information of 841 individuals who were registered with a MS diagnosis. We excluded 67 who had passed away, 69 who had emigrated, and nine who never had an address in the county (tourists, asylum seekers and guest patients). Thus, according to the NPR, there were 696 individuals with MS in Nordland County per January 1, 2017.

**Figure 1:**
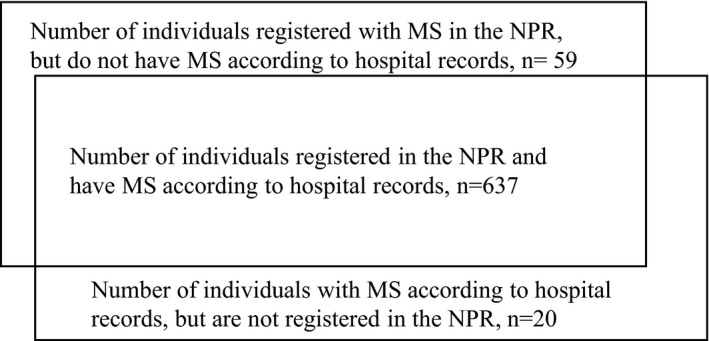
Number of individuals with or without multiple sclerosis (MS) registered or not registered in the Norwegian Patient Registry (NPR)

From hospital record searches, we identified 810 individuals with MS, of whom 608 were living in Nordland County per 1 January 2017. Only 49 of the additional individuals registered with MS in the NPR were TP, and the real number of individuals with MS per January 1, 2017, was thus 657, giving a prevalence of 270.5 per 100,000. Twenty of the individuals with MS identified in medical files were not included in the NPR, and 19 of those were mildly affected by the disease at the latest consultation prior to 2008. In 23 of the 59 individuals registered in the NPR who did not fulfill the diagnostic criteria for MS (Figure 1), later diagnostic work‐up had ruled out MS. In 17 individuals, symptoms or findings were still suspect for MS, but in 19 there was no association to MS. The number of TN was 242,150 (Table 2).

**Table 2:** Cross‐table of individuals with or without multiple sclerosis (MS) registered or not registered in the Norwegian Patient Registry (NPR)

	Confirmed MS according to hospital records		SUM
	Yes	No	
Registered MS in the NPR			
Yes	637	59	696
No	20	242,150	242,170
SUM	657	242,209	242,866

Thus, of those registered with MS in the NPR, 8.5% did not have the disease, and 3.0% of those who have MS were not registered. The sensitivity was 0.97, and the positive predictive value was 0.92. The Cohen's kappa was 0.94.

## 4 DISCUSSION

In Nordland County, 91.5% of those registered with MS in the NPR have a confirmed diagnosis of the disease.

Previous analyses of NPR data for correctness and completeness in stroke in Norway have shown a sensitivity of 96.8% and a specificity of 99.6%, with a positive predictive value of 79.7% (Varmdal et al., 2016). Another study, focusing on intracranial hemorrhage (ICH) in Trøndelag, 8.8% registered with ICH in the NPR showed to be false positive (Øie et al., 2018). In a study of amyotrophic lateral sclerosis (ALS), data from the NPR was validated for Akershus—Hordaland County, showing that 11% of individuals with at least one ALS‐related entry in the NPR had an incorrect diagnosis (Nakken, Lindstrøm, Tysnes, & Holmøy, 2018). These results are in accordance with our findings. Because of the magnitude of the true‐negative value, which is close to the total population in the county, the specificity and the negative predictive value is approximately one. The true‐negative value also highly influences the Cohen's Kappa value, giving a near‐perfect fit.

We found that 3.0% of those who actually have MS are not registered in the NPR. These were individuals that received the diagnosis before 2008 and who had not the diagnosis of MS registered at a hospital since, probably due to a benign course of the disease. This proportion will decrease during time, mainly because all new cases in the NPR are now person identifiable, and to a lesser degree due to the increasing probability that those who were diagnosed with benign disease prior to 2008 eventually will have their diagnosis registered.

Data from the NPR have previously been used in a nationwide prevalence study of MS in Norway, finding a national prevalence of 203 per 100,000 in 2010 (Berg‐Hansen, Moen, Harbo, & Celius, 2014). In that study, unless they used MS specific treatment according to the Norwegian Prescription Database, only individuals with at least two entries of MS in the NPR were included. This was done with the intention to minimize the suspected overestimation of the occurrence.

If we, in the present study, only include those who are registered with MS in the NPR more than once, the overrepresentation of MS in the NPR is reduced from 8.5% to 3.3%. On the other hand, the proportion of those with MS that are not included increases to 6.4%.

### 4.1 Study strengths and limitations

The strength of our study is that the data from the NPR is personally identifiable and that we were able to validate the diagnosis at an individual level. We have a complete overview of the MS population in our county and full access to the hospital records. The limitation with regards to generalizability is that we have validated the NPR with regards to MS in only one county, including <5% of the total Norwegian population.

## 5 CONCLUSION

Data from the NPR give a good estimate of the real prevalence of MS in Nordland County, but nearly one in 10 with a registered diagnosis does not fulfill the diagnostic criteria. Data from the NPR should be combined with data from other sources if more accurate numbers are needed.

## CONFLICT OF INTEREST

The authors have nothing to disclaim related to the topic.

## DISCLAIMER

Data from the Norwegian Patient Registry has been used in this publication. The interpretation and reporting of these data are the sole responsibility of the authors, and no endorsement by the Norwegian Patient Registry is intended or should be inferred.

## Data Availability

The data that support the findings of this study are available from the corresponding author upon reasonable request.
